# External validation of the modified CTP score based on ammonia to predict survival in patients with cirrhosis after TIPS placement

**DOI:** 10.1038/s41598-024-64793-z

**Published:** 2024-06-16

**Authors:** Binlin Da, Wei Wu, Wuhua Guo, Kai Xiong, Chao Chen, Qiao Ke, Moran Zhang, Taishun Li, Jiangqiang Xiao, Lei Wang, Ming Zhang, Feng Zhang, Yuzheng Zhuge

**Affiliations:** 1 Department of Gastroenterology, Nanjing Drum Tower Hospital, Clinical College, Nanjing Medical University, 321#, Zhongshan Road, Nanjing, 210008 Jiangsu China; 2 Department of Gastroenterology, The First Affiliated Hospital of Wenzhou Medical University, Wenzhou, Zhejiang China; 3 Department of Interventional Radiology, Mengchao Hepatobiliary Hospital of Fujian Medical University, Fuzhou, Fujian China; 4 Department of Gastroenterology, The Second Affiliated Hospital of Nanchang University, Nanchang, Jiangxi China; 5 Medical Statistical Analysis Centre, Nanjing Drum Tower Hospital, Clinical College, Nanjing Medical University, Nanjing, Jiangsu China

**Keywords:** Cirrhosis, Transjugular intrahepatic portosystemic shunt, Survival, Prediction model, Risk stratification, Hepatology, Liver cirrhosis

## Abstract

This study aimed to perform the first external validation of the modified Child-Turcotte-Pugh score based on plasma ammonia (aCTP) and compare it with other risk scoring systems to predict survival in patients with cirrhosis after transjugular intrahepatic portosystemic shunt (TIPS) placement. We retrospectively reviewed 473 patients from three cohorts between January 2016 and June 2022 and compared the aCTP score with the Child-Turcotte-Pugh (CTP) score, albumin-bilirubin (ALBI), model for end-stage liver disease (MELD) and sodium MELD (MELD-Na) in predicting transplant-free survival by the concordance index (C-index), area under the receiver operating characteristic curve, calibration plot, and decision curve analysis (DCA) curve. The median follow-up time was 29 months, during which a total of 62 (20.74%) patients died or underwent liver transplantation. The survival curves for the three aCTP grades differed significantly. Patients with aCTP grade C had a shorter expected lifespan than patients with aCTP grades A and B (P < 0.0001). The aCTP score showed the best discriminative performance using the C-index compared with other scores at each time point during follow-up, it also showed better calibration in the calibration plot and the lowest Brier scores, and it also showed a higher net benefit than the other scores in the DCA curve. The aCTP score outperformed the other risk scores in predicting survival after TIPS placement in patients with cirrhosis and may be useful for risk stratification and survival prediction.

## Introduction

Cirrhosis is a well-known destructive disease, and portal hypertension (PHT) is a severe complication of decompensated cirrhosis and carries severe clinical consequences^1^. Transjugular intrahepatic portosystemic shunt (TIPS) has been indicated to be an efficacious approach to decreasing PHT and managing PHT-related complications, such as esophagogastric variceal bleeding (EGVB) and refractory ascites^2,3^. However, the prognosis after TIPS creation is considerably heterogeneous, and it is imperative to use a prognostic risk score to predict survival and risk stratification to better guide post-TIPS management^4^.

Several risk scores have been developed to predict survival and ascertain the individuals who exhibit susceptibility to mortality after TIPS placement. The Child-Turcotte-Pugh (CTP) scoring system was initially established by Child and Turcotte in 1964 and was then revised by Pugh in 1973 to assess the degree of liver dysfunction and the risk in patients undergoing portosystemic shunt surgery, which has been the most widely applied technique thus far^5,6^. Nevertheless, the CTP score has well-known drawbacks. It includes two subjective parameters: ascites and hepatic encephalopathy (HE). Minima HE (MHE) usually has no obvious clinical signs and can only be detected by neuropsychological or neurophysiological testing^7–9^. Ammonia (Amm) is known as a culprit in HE progression. Recently, Amm has been found to exert a crucial prognostic role in liver disease^10,11^. Tranah TH and his colleagues^12^ concluded that plasma Amm was a critical determinant in predicting hospitalization, decompensation events, and even death in cirrhosis patients. Our recent study^13^ developed a modified CTP score based on the plasma Amm (aCTP) score to predict transplant-free survival for patients with decompensated cirrhosis. Since its introduction, no external validation has been completed.

The study aimed to confirm the predictive power of the aCTP score and compare it with other risk scores, including the CTP score, albumin-bilirubin (ALBI) score, model for end-stage liver disease (MELD) score and sodium MELD (MELD-Na) score^14–16^, in predicting transplant-free survival in patients after TIPS creation by using a multicentre database.

## Patients and methods

### Study design

Written informed consent from all subjects included in this study was obtained. For all patients, the guidelines of the 1975 Declaration of Helsinki and Istanbul were followed. All procedures have been approved by the Ethics Committee of The First Affiliated Hospital of Wenzhou Medical University, the Ethics Committee of Mengchao Hepatobiliary Hospital, and the Ethics Committee of The Second Affiliated Hospital of Nanchang University. The study followed the Transparent Reporting of a Multivariable Prediction Model for Individual Prognosis or Diagnosis (TRIPOD) principles^17^.

### Patient selection and methods

Patients from three tertiary hospitals (The First Affiliated Hospital of Wenzhou Medical University, Mengchao Hepatobiliary Hospital, and The Second Affiliated Hospital of Nanchang University) with cirrhosis after TIPS placement between January 2016 and June 2022 were retrospectively screened. The inclusion criteria were as follows: (1) cirrhotic patients undergoing TIPS procedure; (2) patients aged ≥ 18 years; (3) plasma Amm was detected within 72 h before TIPS; and (4) without malignant tumours. The exclusion criteria were as follows: (1) incomplete data; (2) the diameter of the TIPS stent was not 8 mm; and (3) loss to follow-up within 1 year after placement. Two of the three hospitals in this study had TIPS stents with diameters of only 8 mm. The 96 cases of non-8 diameter included 5 cases of 6 mm, 18 cases of 7 mm, 21 cases of 9 mm, 50 cases of 10 mm, and 2 cases of 12 mm, which were small and uneven in number and were not suitable for statistical analysis. So, we excluded the 96 cases of non-8 mm stents. All patients with cirrhosis were treated with causative therapy. Such as, for patients with cirrhosis caused by viral hepatitis, we initiated proactive antiviral therapy immediately upon diagnosis. For those with alcoholic cirrhosis, we have taken proactive measures to encourage and support their abstinence from alcohol. Elective TIPS was performed to treat refractory ascites and prevent EGVB. Refractory ascites is defined as ascites that cannot be mobilised or the early recurrence of which (i.e., after LVP) cannot be satisfactorily prevented by medical therapy^18^. The implementation of TIPS in three cohorts was predominantly centered on the secondary prophylaxis of variceal bleeding. The diagnosis of cirrhosis was made based on medical history and radiological and laboratory data or was confirmed by biopsy. All scoring systems were calculated based on the clinical and laboratory results within 72 h before TIPS implantation (Supplementary Table 1). To facilitate promotion in different hospitals, the ratio of baseline plasma Amm to the laboratory upper limit of normal (Amm-ULN) was calculated rather than using absolute values; it replaced HE in the aCTP scoring system, and Amm-ULN < 1.0 was assigned a score of one, 1.0 ≤ Amm-ULN < 1.4 a score of two, and Amm-ULN ≥ 1.4 a score of three. The scores of other parameters and grades of aCTP scoring system were identical to the CTP score^13^.

### Clinical follow-up and outcomes

After TIPS creation, outpatient follow-up visits were scheduled at 1, 3, 6, and 12 months and then every 1 year or 6 months or as medically necessary. Each outpatient appointment included laboratory testing, abdominal ultrasound, and recurrence assessment for ascites, variceal bleeding, and HE. Patients were followed from the date of TIPS to death or until the current study ended (May 31, 2023). The follow-up of patients undergoing liver transplantation (LT) was terminated at the LT date. The primary endpoints were mortality or LT at each major timepoint after TIPS creation. The secondary outcome was post-TIPS acute decompensation (AD) events, including HE, ascites, and variceal bleeding.

### Statistical analysis methods

Normally distributed data are represented by the mean ± standard deviation (SD), and comparisons were analysed by t test. The nonnormal data were reported as the median (interquartile range), and comparisons were analysed by the Wilcoxon rank sum test. The categorical data are presented as numbers and percentages (%), and comparisons were analysed by the chi-squared test. Based on the Cox proportional hazards model, hazard ratio (HR) for univariable tests and adjusted HR for multivariable tests were calculated with their 95% confidence intervals (CIs). Kaplan–Meier survival curves with log-rank tests were generated. The diagnostic efficacy of the risk scores was assessed with discrimination, calibration, and clinical usefulness. Discrimination was evaluated by the concordance index (C-index) or area under the receiver operating characteristic (ROC) curve (AUROC)^19^. The Brier score and calibration curve were applied to evaluate the calibration ability. A DCA curve was generated to assess the clinical usefulness and net benefit of the scores. Furthermore, the model overall performance was assessed by the R^2^ statistic. R studio (version 4.3.0) was used for all analyses. Statistical significance was defined as a P value < 0.05.

## Results

### Patient characteristics

**Figure 1:**
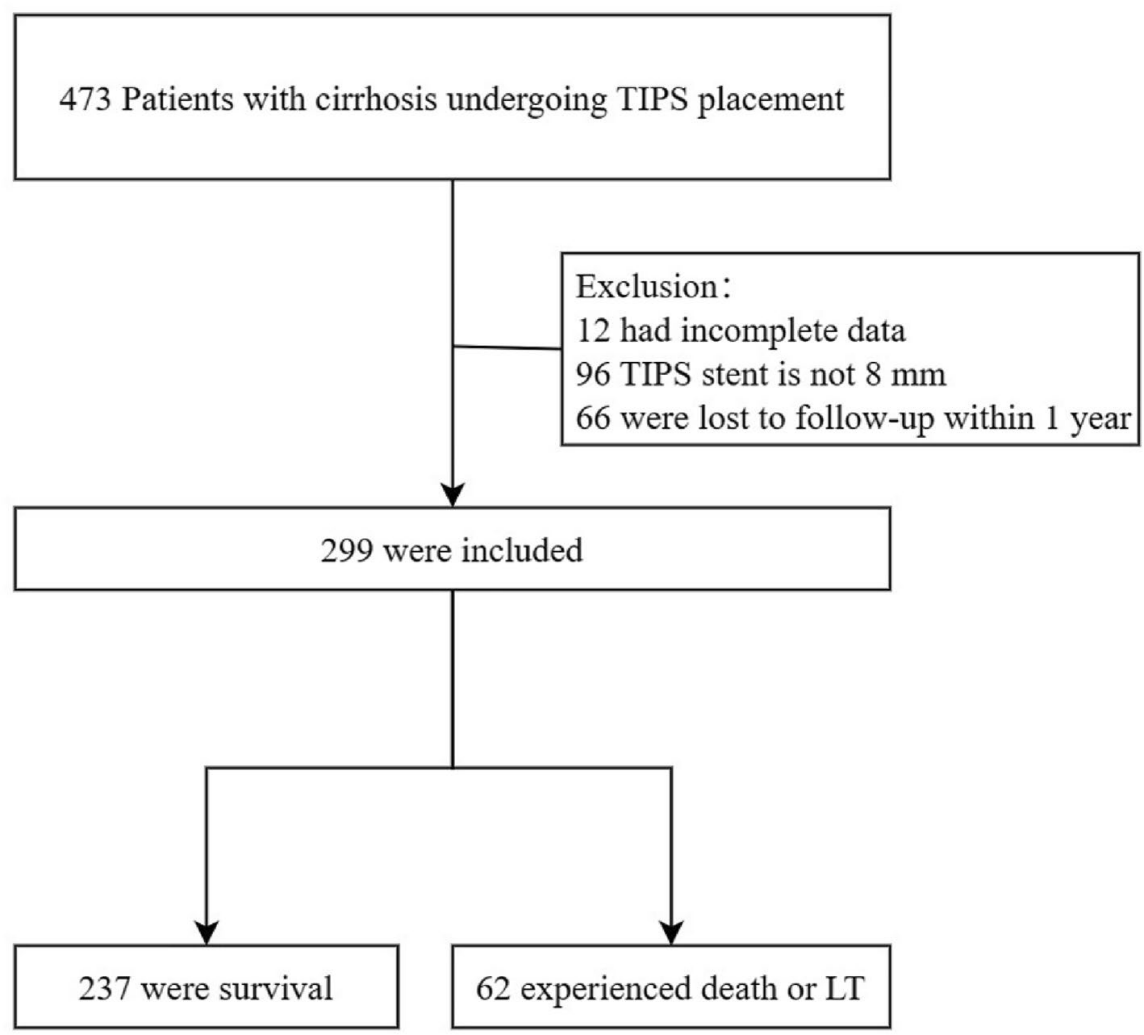
Flowchart of the study.

A total of 473 patients with cirrhosis who underwent TIPS implantation for the prevention of EGVB and treatment of refractory ascites from January 2016 to June 2022 were screened from three hospitals in China. Twelve patients were excluded due to the absence of baseline information, 96 patients were excluded due to the diameter of the TIPS stent, and 66 patients who were lost to follow-up within 1 year were excluded (Fig. 1). In total, 299 patients were included in the analysis. The mean age of the enrolled subjects was 54.22 ± 10.83 years, and 226 (75.59%) were male. The main reasons for liver cirrhosis were hepatitis virus infection (56.86%) and alcohol use (20.40%). TIPS implantation was performed on 273 (91.30%) individuals for variceal haemorrhage and 26 (8.70%) for refractory ascites (Table 1).

**Table 1:** Baseline characteristics of the cohort.

	All patients (n = 299)	Survival group (n = 237)	Death or LT group (n = 62)	P value
Age (years)	54.22 ± 10.83	53.53 ± 10.88	56.85 ± 10.33	0.03
Gender(male/female)	226/73	185/52	41/21	0.052
Causes (virus/alcoholic/others)	170/61/68	137/53/47	33/8/21	0.04
Indication for TIPS (variceal bleeding/ascites)	273/26	218/19	55/7	0.42
Ascites (no/yes)	85/214	77/160	8/54	0.002
HE history (no/1–2/3–4)	291/5/3	232/3/2	59/2/1	0.25
Platelet (×10^9^/L)	71.00 (51.00, 97.00)	71.00 (51.00, 97.00)	70.00 (51.75, 97.25)	0.79
ALT (U/L)	24.00 (16.00, 36.00)	24.00 (16.85, 35.00)	25.00 (14.00, 45.25)	0.46
AST (U/L)	34.00 (24.50, 49.50)	32.00 (24.00, 45.00)	40.50 (28.25, 60.50)	0.01
Albumin (g/L)	30.60 (27.30, 34.75)	31.90 (28.00, 35.10)	28.45 (25.00, 31.30)	< 0.001
Total bilirubin (μmol/L)	22.90 (16.00, 32.00)	21.30 (15.45, 29.85)	28.15 (17.98, 37.28)	0.003
Creatinine (μmol/L)	65.00 (53.00, 78.00)	65.00 (53.00, 78.00)	65.50 (54.00, 81.75)	0.48
PT (s)	16.30 (15.20, 17.85)	16.20 (15.00, 17.70)	17.15 (15.67, 18.75)	0.003
INR	1.34 (1.23, 1.49)	1.32 (1.22, 1.47)	1.42 (1.26, 1.59)	0.01
Na (mmol/L)	138 (136, 141)	138 (136, 141)	137 (134, 140)	0.08
Amm-ULN	0.82 (0.53, 1.20)	0.80 (0.52, 1.11)	1.04 (0.57, 1.45)	0.04
aCTP score	8 (7, 10)	8 (7, 9)	10 (8, 11)	< 0.001
aCTP grade (A/B/C)	59/163/77	57/136/44	2/27/33	< 0.001
CTP score	8 (6, 9)	7 (6, 8)	9 (8, 10)	< 0.001
CTP grade (A/B/C)	76/177/46	70/143/24	6/34/22	< 0.001
ALBI score	− 1.74 ± 0.49	− 1.81 ± 0.48	− 1.50 ± 0.46	< 0.001
MELD score	9.99 (7.99,12.21)	9.87 (7.73,11.70)	11.14 (9.01,15.20)	0.001
MELD-Na score	11.18 (8.84,14.22)	10.59 (8.64,13.20)	14.06 (10.06,18.63)	< 0.001

### Univariable and multivariable analyses

Patients in the cohort had a median follow-up time of 29 (17,42) months and a median survival of 20.5 (7,30) months. Overall, 59 patients died and 3 patients underwent LT during follow-up. Of the 59 patients who died, 51 (86.44%) died from liver-related complications and 8 (13.56%) died from other causes. LT took place 6, 7, and 12 months after TIPS placement. Of the remainder, 237 were still alive and being followed. The post-TIPS overall transplant-free survival rate was 92.64% (277) at 1 year and 86.62% (259) at 2 years. Univariable Cox regression analysis showed that increasing age, alanine aminotransferase (ALT), aspartate aminotransferase (AST), total bilirubin (TB), prothrombin time (PT), international normalized ratio (INR), Amm-ULN, presence of moderate-severe ascites, female sex and decreasing albumin (ALB) and Na significantly increased the risk of post-TIPS death or LT. According to the multivariable analysis, age, sex, TB, moderate-severe ascites, ALT and Amm-ULN were independent mortality or LT risk factors (P < 0.05) (Table 2).

**Table 2:** Univariate and multivariate analysis for post-TIPS mortality or LT.

Predictors	Univariate HR		Multivariate HR	
	HR (95% CI)	P	HR (95% CI)	P
Age	1.03 (1.01, 1.06)	0.008	1.03 (1.00, 1.06)	0.04
Gender (compared with females)				
Male	0.55 (0.32, 0.94)	0.03	0.48 (0.28, 0.85)	0.01
Ascites (compared with without ascites)				
Mild	2.19 (0.96, 5.00)	0.06		
Moderate-severe	3.59 (1.65, 7.70)	0.001	2.75 (1.20, 6.31)	0.02
ALT	1.002 (1.001, 1.003)	0.003	1.01 (1.00, 1.02)	0.048
AST	1.001 (1.000, 1.003)	0.03		
Albumin	0.91 (0.87, 0.96)	< 0.001	0.95 (0.89, 1.00)	0.06
Total bilirubin	1.02 (1.01, 1.03)	< 0.001	1.02 (1.01, 1.03)	0.001
PT	1.15 (1.05, 1.26)	0.004		
INR	3.65 (1.50, 8.9)	0.004		
Na	0.94 (0.90, 0.99)	0.03		
Amm-ULN	1.53 (1.06, 2.21)	0.02	1.58 (1.04, 2.40)	0.03

### Risk stratification of the aCTP score

**Figure 2:**
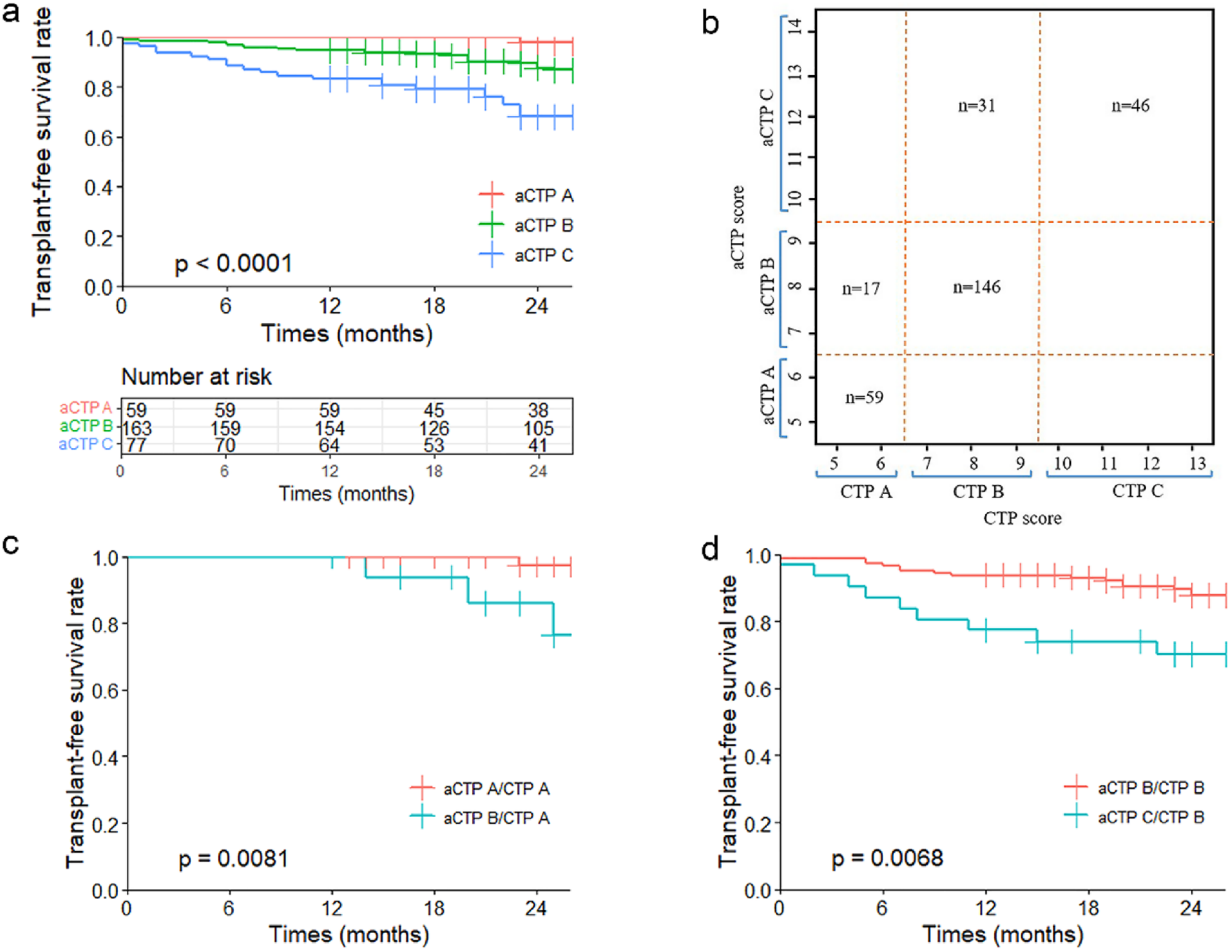
Risk stratification of the aCTP score. (**a**) Kaplan–Meier curves show the transplant-free survival rate for the three grades of aCTP score: patients with an aCTP C grade had a lower transplant-free survival rate than patients with an aCTP A grade and B grade (P < 0.0001). (**b**) The distribution of the CTP and aCTP scores: 76 patients with CTP A were divided as follows: 59 with aCTP A and 17 with aCTP B; 177 patients with CTP B were divided into the following: 146 with aCTP B and 31 with aCTP C; and 46 patients with CTP C were still aCTP C. (**c**) Comparison of the transplant-free survival rates between aCTP A and aCTP B with CTP grade A: the mortality of aCTP B/CTP A was significantly higher than that of aCTP A/CTP A (p < 0.05). (**d**) Comparison of the transplant-free survival rate between aCTP B and aCTP C with CTP grade B: the mortality of aCTP C/CTP B was significantly higher than that of aCTP B/CTP B (p < 0.05).

The survival curves for the three aCTP grades differed significantly, according to our analysis. For grades A (n = 59, 19.73%), B (n = 163, 54.52%), and C (n = 77, 25.75%), the 1-year transplant-free survival rates were 100%, 94.48%, and 83.12%, and the 2-year transplant-free survival rates were 98.31%, 91.41%, and 70.13%, respectively. Patients with an aCTP C grade had a shorter expected lifespan than patients with an aCTP A grade and B grade (P < 0.0001) (Fig. 2a). The distribution of the CTP and aCTP scores is shown in Fig. 2b. 76 patients with CTP A were divided as follows: 59 as aCTP A grade and 17 as aCTP B grade. Significant differences in survival between the aCTP A/CTP A and aCTP B/CTP A groups are shown in Fig. 2c (p < 0.05). From a total of 177 patients with CTP B, 146 were categorized as aCTP B grade and 31 were categorized as aCTP C grade, and the mortality of those categorized as aCTP C/CTP B was significantly higher than that of those categorized as aCTP B/CTP B (p < 0.05) (Fig. 2d).

### The performance of the aCTP score in transplant-free survival

**Figure 3:**
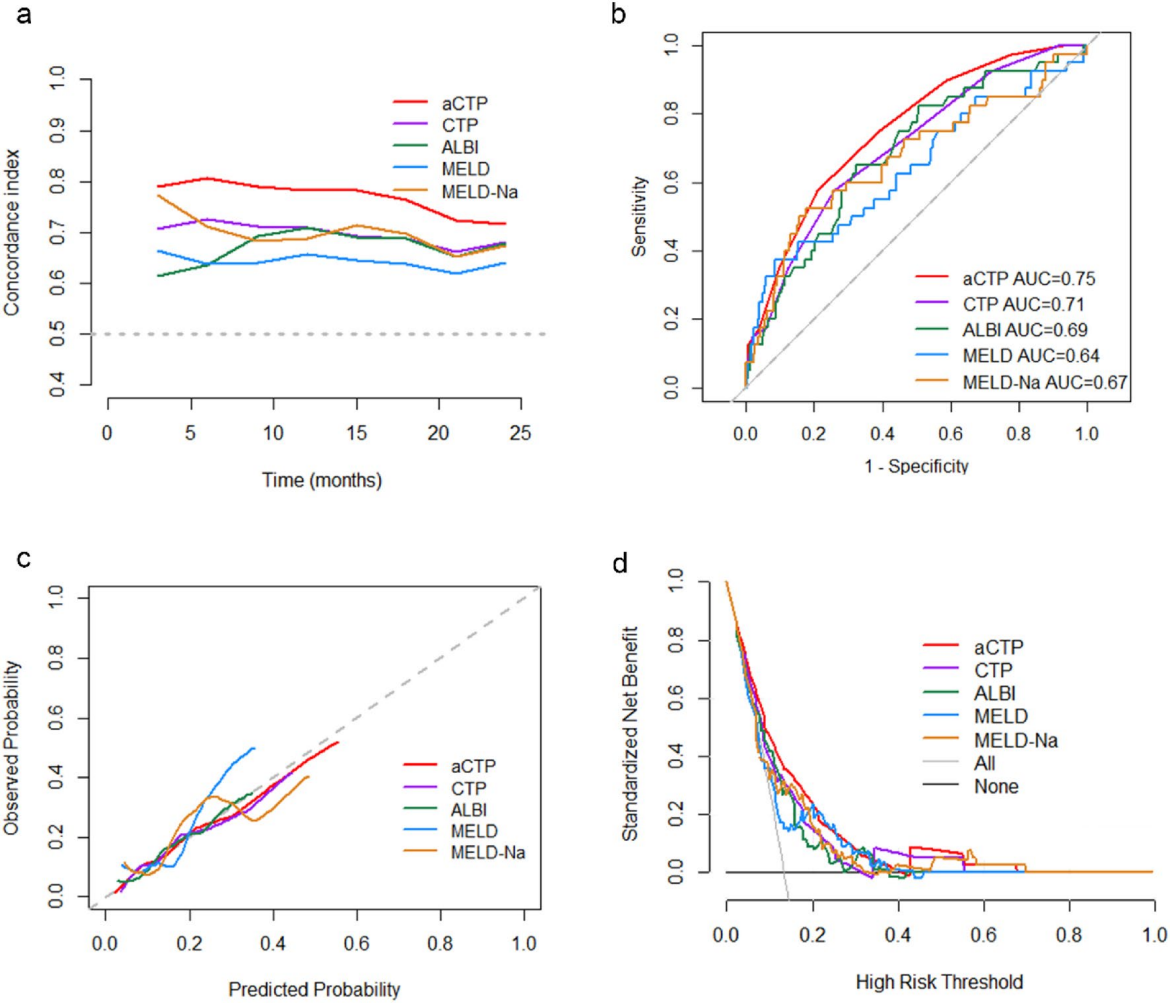
The aCTP score performance in predicting post-TIPS mortality or LT. (**a**) C-index of the aCTP, CTP, ALBI, MELD, MELD-Na scoring systems at predicting post-TIPS mortality or LT during follow-up through 24 months: C-index of the aCTP score was greater than that of the other scores at predicting transplant-free survival at each time point of follow-up. (**b**) ROC curve of the aCTP, CTP, ALBI, MELD, and MELD-Na scoring systems for 2-year transplant-free survival: the AUROC of aCTP was greater than that of the other scores. (**c**) Calibration plot comparing the observed and predicted mortality or LT based on the aCTP, CTP, ALBI, MELD, and MELD-Na scoring systems in 2 years: The superior agreement between the observed and predicted mortality or LT by the aCTP score and ALBI score was revealed compared to the other scores. (**d**) DCA curve of the aCTP, CTP, ALBI, MELD, and MELD-Na scores in predicting 2-year transplant-free survival: the standardized net benefit of aCTP was higher than that of the other scores.

Individuals who died or experienced LT during the follow-up period had significantly higher aCTP scores than those who lived (10 vs. 8; P < 0.001). Among the risk scores (Table 3), the discriminative performance in predicting the 3-month transplant-free survival rate was the highest for the aCTP score. At later time points, the aCTP score also demonstrated preferred discrimination (Fig. 3a). The ROC curve (Fig. 3b) in predicting the 2-year transplant-free survival rate was the highest for the aCTP score (AUROC 0.75), followed by CTP (AUROC 0.71), ALBI (AUROC 0.69), MELD-Na (AUROC 0.67), and MELD (AUROC 0.64). The AUROC of aCTP had statistical differences with that of CTP and MELD (P values 0.035 and 0.033, respectively) using Z test, but no statistical differences with that of ALBI and MELD-Na (P values > 0.05). The calibration curve revealed that the aCTP and ALBI scores showed a higher agreement between the observed and predicted 2-year transplant-free survival compared with the other risk scores (Fig. 3c). The lower Brier scores for the aCTP score from 3 to 24 months compared to the other risk scores also suggested superior calibration (Table 3). The DCA curve for predicting 2-year transplant-free survival showed that the aCTP score had a higher net benefit than the other scores (Fig. 3d). For overall performance, the aCTP score had the highest R^2^ value at every major time point (such as at 3 months, R^2^ value of 12.3% for aCTP vs. 6.9% for CTP, 6.5% for MELD-Na, 3.7% for MELD and 2.2% for ALBI) (Table 3).

**Table 3:** Performance of the risk scores in predicting post-TIPS mortality or LT.

Scoring system	C-Index^a^				Brier-score^b^				R^2^ (%)			
	3 m	6 m	12 m	24 m	3 m	6 m	12 m	24 m	3 m	6 m	12 m	24 m
aCTP	0.79	0.81	0.79	0.75	0.025	0.034	0.064	0.102	12.3	14.1	16.9	18.4
CTP	0.71	0.69	0.72	0.71	0.026	0.035	0.065	0.107	6.9	5.5	9.5	12.5
ALBI	0.61	0.60	0.72	0.69	0.026	0.035	0.065	0.110	2.2	1.8	11.1	8.8
MELD	0.67	0.61	0.67	0.64	0.026	0.035	0.066	0.109	3.7	1.5	6.2	6.9
MELD-Na	0.78	0.67	0.69	0.66	0.026	0.035	0.064	0.110	6.5	2.9	8.6	7.2

^a^Represents a measure of discrimination whereby values closer to 1 indicate better discriminative ability.

^b^Represents a measure of calibration whereby values closer to 0 indicate better calibration ability.

### Performance of the aCTP score in predicting post-TIPS HE and ascites

A total of 124 (41.47%) patients experienced at least one post-TIPS AD event, including HE (n = 94), ascites (n = 33), and variceal bleeding (n = 27). Furthermore, we assessed the efficacy of the aCTP score in predicting these events. We compared only three models, including the aCTP, CTP, and ALBI models, because of their higher discrimination (C-statistics > 0.60). Among the three models (Supplementary Table 2), the discriminative performance in predicting HE events was highest for the aCTP score (AUROC, 0.65), followed by the ALBI score (AUROC, 0.61) and CTP score (AUROC, 0.61). A preferable discrimination was seen for aCTP score for ascites. The lower Brier scores in post-TIPS HE and ascites events for the aCTP score compared to the other risk scoring systems also demonstrated improved calibration (Supplementary Table 2). Meanwhile, the aCTP score had the highest R^2^ value in post-TIPS HE and ascites events (Supplementary Table 2). The predictive ability of all three models for variceal bleeding was poor.

## Discussion

The study found that the independent risk factors for mortality were age, sex, TB, moderate-severe ascites, ALT, and Amm-ULN in patients with cirrhosis undergoing TIPS. The aCTP score showed superior discrimination, calibration, and overall performance than the CTP, ALBI, MELD, and MELD-Na scores for the prediction of post-TIPS transplant-free survival across 2-year intervals. It also showed a more inspiring predictive power for AD events after TIPS creation than the other scores, especially for HE.

In traditional scoring systems, the CTP score is currently the most frequently applied score in clinical practice. For example, BAVENO VII recommended CTP B > 7 points with active variceal bleeding or C < 14 points as an indication for early TIPS (pre-emptive TIPS)^6^. However, CTP includes a subjective parameter, HE, that is challenging to quantify clinically and detect early. The HE variable in the CTP score completely depends on the physician’s evaluation, which contributes to bias and inconsistency in the results.

The metabolism and elimination of plasma Amm mainly rely on the urea cycle and glutamine synthetase. Liver dysfunction in cirrhosis reduces these two clearance pathways and then induces hyperammonaemia, which has been implicated in some cirrhosis-related complications. Studies on humans and animals have shown that elevated Amm levels can exacerbate sarcopenia^20^, provoke immunological dysregulation, and even have direct hepatotoxic effects^21^, despite being most known for its involvement in HE. A prospective study was conducted in 2022 to appraise the relevance between plasma Amm and adverse consequences in stable cirrhosis patients and concluded that plasma Amm played a key role in predicting which patients would require hospitalization, experience liver-associated complications, and even die^12^. The aCTP score was then developed by our team^13^, and it was shown to be better than the CTP score in predicting survival in patients with decompensated cirrhosis. However, the study only included internal data, so this study was undertaken to validate its generalization performance.

In the present study, we found that the aCTP score reversed the deterioration of the CTP score and reclassified approximately 1/6 of patients. The time-dependent C-index of the aCTP score in predicting post-TIPS transplant-free survival varied from 0.75 to 0.81, which was higher than the CTP score; the calibration ability and clinical usefulness of the aCTP score were also superior to the CTP score, which is in line with the data from the development and internal validation cohorts^13^. At the same time, the time-dependent C-index and R^2^ of aCTP were also obviously higher than those of the MELD, MELD-Na, and ALBI scoring systems across 2-year intervals, and the Brier score of aCTP was lower than that of the other scores, indicating superior discrimination, calibration, and overall ability.

In 2001, the MELD score was first established for predicting poor survival in TIPS patients, especially within 3 months, and then it was modified in 2001 by multiplying each coefficient by ten and was named Mayo MELD^14,22^. A study^23^ verified a cutoff for the Mayo MELD score of > 18 and discovered that the 3-month mortality after TIPS creation was apparently lower in patients with MELD scores < 18 than in those with MELD scores ≥ 18. During the process of advanced cirrhosis, hyponatremia is common and closely related to the advancement of ascites, hepatorenal syndrome and even death. Biggins et al.^24^ developed the MELD-Na score by including serum sodium in the MELD score, which showed improvement for the survival prediction in LT candidates with terminal liver disease in comparison with the MELD score. However, some studies demonstrated that MELD was superior in predicting 30-mortality after TIPS creation than MELD-Na^25,26^. Our study suggested that the MELD-Na score exhibited a higher C-index and R^2^ than the MELD score in predicting transplant-free survival, which revealed that MELD-Na performed with superior discriminative and overall performance than the MELD score, but two, owing to the similar Brier score, revealed similar calibration ability. The discriminative accuracy of these scores, especially the MELD-Na score, diminishes over time after TIPS creation, as in previous studies^27,28^.

The ALBI grade was established in 2015 to assess the liver function of individuals with hepatocellular carcinoma^16^. A retrospective study conducted by Ronald et al. revealed that the MELD score had a superior ability to predict 30-day and overall mortality than the ALBI grade and AIBI score. In this study, ALBI discrimination performance outperformed the MELD score and was similar to that of the CTP score. In 2021, Bettinger et al.^29^ created a new score in a German cohort named the Freiburg index of post-TIPS survival (FIPS) and verified that it outperformed the CTP, MELD, and MELD-Na scoring systems in identifying high-risk patients with poor prognosis undergoing TIPS placement. Nevertheless, in an external validation study^30^, the FIPS score did not perform better than MELD-Na in an era from 2014 to 2020 and showed poorer calibration than the CTP, MELD, and MELD-Na scoring systems. Although the data were not disclosed, we found that the FIPS score did not work well in our cohort.

The present study had several potential limitations. First, many patients had to be excluded because of baseline plasma Amm deficiency, which resulted in potential bias in patient selection. Second, although this was a multicentre study, the overall sample size was still limited, and it was impossible to disregard data bias. Third, the study was a retrospective study with all the inherent limitations that it entails. Fourth, this study had a high loss of follow-up rate due to multi-center, retrospective study, and difficult follow-up. The potential for bias due to loss to follow-up is a critical consideration. If the reasons for dropout are related to the outcomes of interest, this could lead to systematic errors in the estimated effects of the interventions or exposures under study.

In brief, the aCTP scoring system showed superior prognostic performance compared with other scoring systems in predicting both short- and long-term transplant-free survival and post-TIPS AD events. However, further investigations of prospective cohorts with larger sample sizes are warranted.

### Supplementary Information

Supplementary Tables.

## Data Availability

The datasets used during the current study available from the corresponding author on reasonable request.
