# Supplementary material for: External validation of the modified CTP score based on ammonia to predict survival in patients with cirrhosis after TIPS placement
Source: Sci Rep. 2024 Jun 16;14:13886. doi: 10.1038/s41598-024-64793-z (PMC11180650; doi:10.1038/s41598-024-64793-z)
Supplement: Supplementary file 1 — Supplementary Tables. [file 41598_2024_64793_MOESM1_ESM.docx]

**Supplementary Table 1** Formulas for Calculating Model for End-Stage Liver Disease (MELD), Sodium MELD (MELD-Na) and Albumin Bilirubin (ALBI) Scores; components for Child-Turcotte-Pugh (CTP) and CTP score adjusted by plasma ammonia (aCTP scores)

| Scores | Formula/components |
| --- | --- |
| CTP (1973) ^5^ | HE (grade), ascites (grade), bilirubin (μmol/L), albumin (g/L), INR |
| aCTP (2023) ^13^ | Amm-ULN (grade), ascites (grade), bilirubin (μmol/L), albumin (g/L), INR |
| Mayo MELD (2001) ^21^ | 9.6 × LN (creatinine mg/dL) + 3.8 × LN (bilirubin mg/dL) + 11.2 × LN (INR) + 6.4*(etiology: 0 if cholestatic or alcoholic, 1 otherwise) |
| MELD-Na (2006) ^23^ | MELD + 1.59 × (135 – Na mmol/L) with maximum and minimum Na of 135 and 120 mmol/L, respectively |
| ALBI (2015) ^16^ | 0.66 × log10 (bilirubin μmol /L) – 0.085 × albumin in g/L |

HE, hepatic encephalopathy; INR, international normalized ratio. Amm-ULN, plasma ammonia / upper limit of the normal

**Supplementary Table 2** Performance of the risk scores in predicting post-TIPS HE and ascites

| Scoring | C-Index | | Brier-Score^a^ | | R^2^(%)^b^ | |
| --- | --- | --- | --- | --- | --- | --- |
| System | HE | ascites | HE | ascites | HE | ascites |
| aCTP | 0.65 | 0.63  0.59  0.53 | 0.205 | 0.096  0.097  0.098 | 7.4 | 4.3  2.2  0.2 |
| CTP | 0.61 |  | 0.209 |  | 4.3 |  |
| ALBI | 0.61 |  | 0.210 |  | 4.0 |  |

CTP, Child-Turcotte-Pugh; aCTP, modified CTP based on plasma ammonia; ALBI, albumin-bilirubin; HE, hepatic encephalopathy

^a^Represents a measure of discrimination whereby values closer to 1 indicate better discriminative ability

^b^Represents a measure of calibration whereby values closer to 0 indicate better calibration ability
